# What the G.P. Wants from a Casualty Department

**Published:** 1955-09

**Authors:** 


### WHAT THE G.P. WANTS FROM A CASUALTY DEPARTMENT

BY

A GENERAL PRACTITIONER

*Casualties coming in direct.* The general practitioner will expect that cases will be admitted when necessary. When the injury is such that it can be dealt with and the patient discharged, the doctor expects to have a note sent with the patient from the casualty officer briefly stating the injury, X-ray findings, what has been done, and the line of treatment recommended. Where the seriousness of the case demands it, the doctor should be informed about it by 'phone.

It should be unnecessary for the doctor to have to write a letter referring his patient to Out-Patients, if that patient has sought advice himself at the Casualty department.

*Casualties referred by the family doctor.* If the injury can be dealt with at once, the G.P. expects a report on lines similar to that given for direct casualties. If he has expressed a wish for the case to be detained or admitted the G.P. expects that this will be done. No-one below the status of a registrar should take the decision to refuse admission in such circumstances. On the other hand often the single visit to Casualty would suffice, and chemotherapy, dressings and removal of stitches could be left to the family doctor, who has the district nurse to call on. This would ease things at the department, without unduly loading the doctors individually. In these days of litigation, many cases are referred by practitioners for X-Ray examination to protect himself against legal action, and a great deal of unnecessary radiography has to be accepted. Prompt reports on the wet-films are therefore much appreciated.

*Septic fingers and hands.* Finger sepsis can lead to serious and prolonged disability, and the family doctor is glad to have available such a service with adequate anaesthesia and skilled surgery.

*Cold minor surgery,* and so forth. Individual doctors vary in their desire to engage in minor surgery such as wens, and lipomata, and since the financial barrier to the hypochondriac's access to the doctor has been removed, time for minor surgery is hard to find, and some doctors and their patients are grateful to have easy access to Casualty for this sort of treatment.

The time spent in the casualty department should be reasonable. Patients tend to complain when they have no ground to do so and here the G.P. can help by explaining to patients why X-Rays and treatment take time.

*Accommodation.* Patients should have proper privacy for undressing and should be spared any unpleasant experiences such as seeing other people's wounds being dressed.

The family doctor has at his disposal in Bristol a casualty department at the Royal Infirmary which meets his requirements very well. This seems to be attributable to the appointment by the Board of Governors of a casualty surgeon of consultant status. The lack of a casualty department in the General Hospital is still felt; an eight- or twelve-hour service at the B.G.H. would be a help to south Bristol and would relieve pressure at the B.R.I. For an industrial city of over half a million, the present casualty departments at B.R.I., Southmead and Cossham-Frenchay hardly meet the need; and the Regional Board should not continue to leave unused the commodious department at the B.G.H.

